# Fabrication and Evaluation of Polyhydroxyalkanoate-Based Nanoparticles for Curcumin Delivery in Biomedical Applications

**DOI:** 10.3390/molecules30061216

**Published:** 2025-03-08

**Authors:** Fawzia Sha’at, Dana Miu, Mihaela Carmen Eremia, Georgeta Neagu, Adrian Albulescu, Radu Albulescu, Mihaela Deaconu, Mariana Gratiela Vladu, Ramona-Daniela Pavaloiu

**Affiliations:** 1 National Institute for Chemical-Pharmaceutical Research & Development—ICCF, Bucharest 112 Vitan Avenue, 3rd District, 031299 Bucharest, Romania; fawzya.shaat@gmail.com (F.S.); dana.miu92@gmail.com (D.M.); mihaelaceremia@yahoo.com (M.C.E.); getabios@yahoo.com (G.N.); adrian.albulescu@virology.ro (A.A.); radu_a1@yahoo.com (R.A.); marianagratielavladu@gmail.com (M.G.V.); 2 Faculty of Chemical Engineering and Biotechnologies, University Politehnica of Bucharest, 1-7 Gheorghe POLIZU St., 011061 Bucharest, Romania; mihaela_deaconu@yahoo.com; 3 Department of Molecular Virology, Stefan S. Nicolau Institute of Virology, Mihai Bravu Av. nr. 285, 3rd District, 030304 Bucharest, Romania

**Keywords:** polyhydroxyalkanoates, biopolymeric nanoparticles, biomedical applications, biocompatibility, cytotoxicity, skin irritation

## Abstract

This study investigates the fabrication and characterization of polymeric nanoparticles based on polyhydroxyalkanoates (PHAs) loaded with curcumin for biomedical applications. PHAs, biodegradable and biocompatible polymers, were synthesized via bacterial fermentation and used to encapsulate curcumin using the nanoprecipitation method. The resulting nanoparticles were characterized for their particle size, polydispersity index, and encapsulation efficiency, achieving high entrapment rates (above 80%) and nanometric size distribution. Stability assessments demonstrated prolonged structural integrity under storage conditions. In vitro release studies conducted in phosphate-buffered saline at pH 5 and 7.4 revealed sustained drug release profiles. Biocompatibility and cytotoxicity assays using human astrocytes and fibroblasts confirmed the nanoparticles’ safety, while antiproliferative tests on glioblastoma and colon cancer cell lines indicated potential therapeutic efficacy. Additionally, skin irritation and corrosion tests using the EpiDerm™ model classified the formulations as non-irritant and non-corrosive. These findings suggest that PHA-based nanoparticles offer a promising nanocarrier system for curcumin delivery, with potential applications in cancer treatment and regenerative medicine. Future research should focus on optimizing the formulation and evaluating in vivo therapeutic effects.

## 1. Introduction

Polymeric nanoparticles have emerged as a promising platform for drug delivery systems due to their ability to enhance the bioavailability, stability, and targeted delivery of therapeutic agents [1,2,3]. Among the various biodegradable polymers, polyhydroxyalkanoates (PHAs) stand out as an eco-friendly and versatile class of biopolymers. PHAs are a class of polyesters produced by bacterial fermentation using various microorganisms like *Methylobacterium* sp., *Azotobacter* sp., *Aspergillus eutrophus, Cupriavidus necator*, *Rhodobacter sphaeroides, Pseudomonas* sp., *Hydrogenophaga pseudoflava, Haloferax mediterranei, Saccharophagus degradans, Halomonas* sp., *Azohydromonas* sp., *Comamonas* sp., *Dechloromonas* sp., *Aeromonas* sp., etc. [4,5,6]. PHAs are biocompatible and non-toxic, have adjustable mechanical properties, and are capable of degrading into natural byproducts, making them highly suitable for biomedical applications [7,8,9]. These characteristics make PHAs a valuable material for various biomedical applications, such as carriers for pharmaceutical active ingredients and genes, vaccine delivery, bio-imaging, biodegradable implants, and tissue engineering [7,8,9,10,11,12,13].

Curcumin, a bioactive compound derived from Curcuma longa, has garnered significant attention due to its multifaceted pharmacological properties, including antioxidant, anti-inflammatory, antimicrobial, anticancer, and neuroprotective effects. However, its clinical application is hindered by poor aqueous solubility, rapid metabolism, and low bioavailability. To address these challenges, curcumin has been encapsulated into various drug delivery systems, such as liposomes, inorganic nanoparticles, polymeric nanoparticles, micro/nanoemulsions, etc. [14,15]. Encapsulation of curcumin within polymeric nanoparticles offers a viable strategy to overcome these limitations, enhancing its stability and therapeutic potential. Several natural biodegradable polymers have been used to encapsulate curcumin within polymeric nanoparticles, like chitosan [16,17], alginate [18,19,20], starch [21], pullulan [22]. While many biodegradable polymers have been employed for curcumin encapsulation, limited studies have explored the use of PHAs for this purpose. A noteworthy study by Senthilkumar et al. (2017) used polyhydroxybutyrate (PHB), obtained from *Pseudomonas aeruginosa*, to synthesize curcumin-loaded nanoparticles with size ranges between 300 and 500 nm, smooth and spherical morphology, and demonstrated sustained drug release for over five hours [23]. The curcumin released from the PHB nanoparticles retained its bioactivity, displaying antibacterial properties when acetic acid was employed as the solvent.

The innovative aspect of this study lies in the development of polymeric nanoparticles using polyhydroxyalkanoates (PHAs) with tailored compositions—polyhydroxyheptanoate (PHH), polyhydroxyoctanoate (PHO), and polyhydroxynonanoate (PHN)—produced by bacterial fermentation of *Pseudomonas putida*, representing a significant advancement in the field of biopolymer-based drug delivery systems. The PHH, PHO, and PHN are medium-chain-length PHAs and offer distinct advantages over a short-chain-length PHA like PHB, including having improved mechanical flexibility and lower crystallinity, making them more flexible and elastomeric and with more tunable degradation rates [24]. Additionally, by leveraging the PHA compositions, this study provides a more versatile platform for nanoparticle-based drug delivery, potentially overcoming limitations associated with PHB, such as its brittleness and narrow processing window. Unlike previous studies that primarily utilized polyhydroxybutyrate (PHB), this pioneering work explores a broader range of PHA compositions, allowing for enhanced tunability of nanoparticle properties such as size, stability, and drug encapsulation efficiency.

Therefore, this study aims to develop and characterize polymeric nanoparticles based on PHAs loaded with curcumin for biomedical applications. The nanoparticles were synthesized using the nanoprecipitation technique, ensuring nanometric size, high encapsulation efficiency, and stability. Their performance as a drug delivery system was evaluated through comprehensive characterization, including entrapment efficiency, size distribution, and stability assessments using spectrophotometric and dynamic light scattering techniques. Additionally, in vitro release studies were conducted under two physiological media, with neutral (pH 7.4) and acidic (pH 5) conditions, to simulate different biological environments. To assess their biocompatibility and therapeutic potential, the nanoparticles were tested for cytotoxicity on normal human astrocytes (NHAs) and fibroblasts, as well as for antiproliferative effects on glioblastoma (U87 MG) and colon cancer (Caco-2) cell lines. Furthermore, skin irritation and corrosivity were evaluated using the EpiDerm™ in vitro skin model, ensuring their safety for potential dermal applications. By utilizing PHAs with tailored compositions obtained from *Pseudomonas putida* fermentation, this study provides a novel approach for the development of biodegradable and efficient drug delivery systems. The findings highlight the potential of PHA-based nanoparticles in enhancing the stability, bioavailability, and therapeutic efficacy of curcumin, paving the way for future biomedical applications.

## 2. Results and Discussion

### 2.1. Characteristics of Polymeric Nanoparticles Based on Polyhydroxyalkanoates

Curcumin-loaded polymeric nanoparticles were effectively synthesized using the nanoprecipitation technique, which is known for its reproducibility, cost-efficiency, and effectiveness. The polymer matrices, composed of polyhydroxyalkanoates (PHAs), were obtained through microbial fermentation by *Pseudomonas putida* ICCF 391. Three distinct PHAs were utilized—polyhydroxyheptanoate (PHH), polyhydroxyoctanoate (PHO), and polyhydroxynonanoate (PHN)—each exhibiting unique hydroxyacid compositions. Specifically, PHH contained the highest proportion of 3-hydroxyheptanoic acid (87.46%), PHO predominantly featured 3-hydroxyoctanoic acid (85.93%), while PHN was rich in 3-hydroxynonanoic acid (73.28%). These compositional variations significantly impacted the properties and functionality of the resulting nanoparticles.

The characteristics of the nanoparticles, such as entrapment efficiency (EE), particle size, and polydispersity index (PDI), are presented in Table 1. The encapsulation efficiency was consistently high with values ranging from 80.41 to 84.35%. In the nanoprecipitation method, a high affinity between the active pharmaceutical ingredient (API) and the polymer for the same solvent typically leads to efficient encapsulation. In our study, both curcumin and PHAs exhibited strong affinity for acetone, the chosen organic solvent, which facilitated the formation of polymeric nanoparticles with superior entrapment efficiency. During nanoprecipitation, the evaporation of acetone induces supersaturation of PHAs in the aqueous phase, leading to its precipitation and the encapsulation of curcumin molecules within the polymeric matrix. The rapid diffusion of the organic solvent into water further promotes the formation of small, uniformly distributed nanoparticles. This approach is particularly notable for its simplicity, reproducibility, and ability to generate high-quality nanoparticles, making it a valuable method in drug delivery and nanomedicine. The size and homogeneity of nanoparticles are key features influencing their circulation time and cellular uptake. Homogeneity is assessed by measuring the polydispersity index; monodisperse systems have a polydispersity index value bellow 0.1, while systems with polydispersity index values higher than 0.7 are considered polydisperse systems. The particle size and polydispersity index of nanoparticles loaded with curcumin were determined by dynamic light scattering (DLS) technique. As shown in Table 1, particle sizes ranged from 307.5 to 315 nm, with PDIs below 0.29, indicating moderate homogeneity. These results are consistent with previous studies, such as those involving polyhydroxybutyrate (PHB) nanosystems loaded with curcumin, which exhibited similar particle sizes and high entrapment efficiencies [25,26]. Similarly, prior research on microbial polymer-based nanoparticles has shown analogous findings. For example, pullulan acetate nanoparticles loaded with epirubicin achieved drug loading efficiencies of up to 64.8% and particle sizes between 185.7 and 423 nm [27]. Likewise, pullulan acetate nanoparticles incorporating paclitaxel demonstrated particle sizes of 253.0 ± 65.5 nm, with drug contents reaching 13.1% [28]. Also, we obtained smaller-sized particles for curcumin-loaded nanoparticles compared to the study conducted by Senthilkumar et al. (2017) that used polyhydroxybutyrate (PHB) [23].

The stability of nanoparticles based on PHAs loaded with curcumin was evaluated by storing them in amber glass vials at 4 °C for three months. The nanoparticles were suspended in PBS at pH 7.4 without any stabilizers. The entrapment efficiency was determined at 1, 2, and 3 months for samples kept at 4 °C. The results demonstrated that the nanoparticles maintained structural integrity and entrapment efficiency for up to three months when maintained at 4 °C. Additionally, minimal drug loss (<2% after three months) and the absence of aggregation, as assessed by DLS measurements conducted at each time point, confirmed their suitability for prolonged storage (Table 2 and Table 3).

### 2.2. In Vitro Release Profiles of Curcumin from PHA-Based Nanoparticles

**Figure 1:**
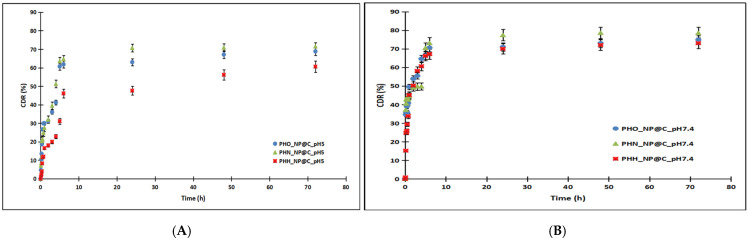
The release profiles of curcumin from nanoparticles based on polyhydroxyalkanoates in PBS 0.1 M pH 5 (**A**) compared to PBS 0.1 M pH 7.4 (**B**). The release followed the trend PHH < PHO < PHN in both media, indicating a composition-dependent effect on drug release.

The in vitro release profiles of curcumin from PHA-based nanoparticles were investigated under two physiological conditions: a neutral environment (PBS, pH 7.4) and a mildly acidic environment (PBS, pH 5). Release rates were significantly higher in the neutral medium compared to the acidic one, with a distinct initial burst release observed in both cases (Figure 1). This phenomenon, attributed to the rapid release of surface-adsorbed curcumin, was more pronounced in PBS (pH 7.4), with 29.40%, 35.73%, and 43.96% of curcumin released within 30 min for PHH, PHO, and PHN, respectively, compared to 11.50%, 26.70%, and 21.40% in PBS (pH 5). Cumulative release after 72 h reached maximum values of 73.01% (PHH), 74.84% (PHO), and 78.51% (PHN) in PBS (pH 7.4) and 60.60%, 68.80%, and 71.56% in PBS (pH 5). The slower release observed in the acidic medium is likely due to reduced polymer swelling and slower diffusion. The release profiles suggest that curcumin release is influenced by the molecular weight (M_W_) of the PHA polymer and the curcumin content in the nanoparticles, with the rank order of release being PHH < PHO < PHN in both media. The molecular weight of PHH, PHO, and PHN is 110.5 kDa, 141.8 kDa, and 165 kDa, respectively, with higher M_W_ generally leading to more rigid polymer matrices that slow drug diffusion. This aligns with the observed trend, where PHH (lowest M_W_) had the slowest release, while PHN (highest M_W_) showed the fastest release. Additionally, curcumin content slightly varied among formulations (PHH_NP@C: 4.12 mg, PHO_NP@C: 4.21 mg, PHN_NP@C: 4.25 mg). Although these differences are small, a higher drug load in PHN may contribute to its relatively higher release, as a greater concentration gradient can enhance diffusion. However, the dominant factor appears to be the PHA molecular weight, as the trend (PHH < PHO < PHN) is consistent across both pH conditions, suggesting that polymer characteristics primarily govern release behavior. These findings indicate that by modulating PHA M_W_, it is possible to tailor the drug release rate, which is crucial for optimizing formulations for sustained or controlled drug delivery applications.

Curcumin release mechanism: kinetic modeling and evaluation of release profiles

Seven kinetics models, such as zero-order, first-order, Korsmeyer–Peppas, Higuchi, Hixson–Crowell, Weibull, and Baker–Lonsdale, were applied to predict the curcumin release profile (Equations (1)–(7)):

(1) $$Q_{t}=K_{0}\times t\;(\mathrm{Zero}\text{-}\mathrm{order}\;\mathrm{model})$$

(2) $$Q_{t}=100\times (1-e^{-K_{1}\times t})\;(\mathrm{First}\text{-}\mathrm{order}\;\mathrm{model})$$

(3) $$Q_{t}=K_{KP}\times t^{n}\;(\mathrm{Korsmeyer}\text{-}\mathrm{Peppas}\;\mathrm{model})$$

(4) $$Q_{t}=K_{H}\times t^{0.5}\;(\mathrm{Higuchi}\;\mathrm{model})$$

(5) $$Q_{0}^{1/3}-Q_{t}^{1/3}=K_{S}\times t\;(\mathrm{Hixson}\text{-}\mathrm{Crowell}\;\mathrm{model})$$

(6) $$m=1-exp[\frac{-t^{b}}{a}]\;(\mathrm{Weibull}\;\mathrm{model})$$

(7) $$\frac{3}{2}\times [1-(1-Q{)}^{2/3}]-Q=K_{BL}\times t\;(\mathrm{Baker}\text{-}\mathrm{Lonsdale}\;\mathrm{model})$$

where *t* is the time, *Q_t_* is the amount of active substance released at time *t*, *Q*_0_ is the initial amount of active substance, *K_1_* is the release constant for first-order kinetics, *K*_0_ is the release constant for zero-order kinetics, *K_H_* represents the Higuchi constant, *K_KP_* is the Korsmeyer–Peppas constant, *n* is the exponential factor, *Ks* is the Hixson–Crowell constant, *a* and *b* are Weibull parameters (time constant, respectively, shape parameter), *K_BL_* is the Baker–Lonsdale release rate constant, *m* is the amount of drug released, and for the Weibull model, *Q_t_* = 100%*m.

A reliable prediction model is defined by an R^2^ value close to 1, along with a lower root mean square error (RMSE) and Akaike information criterion (AIC). The curcumin release kinetics were evaluated using KinetDS 3 software [29].

The parameters (R^2^, AIC, and RMSE) for the zero-order, first-order, Korsmeyer–Peppas, Higuchi, Hixson–Crowell, Weibull, and Baker–Lonsdale models are presented in Table 4.

The Korsmeyer–Peppas and Weibull models best matched the experimental data, as indicated by the high correlation coefficients (R^2^ values > 0.93) and low AIC and RMSE values. The Korsmeyer–Peppas and Weibull parameters are provided in Table 5. These models effectively describe drug release from polymeric systems, particularly when multiple mechanisms (e.g., diffusion, swelling, and erosion) contribute to the release profile. The Korsmeyer–Peppas model is widely used to characterize drug release from swelling and eroding polymeric systems. In this model, the release exponent (*n*) determines the underlying mechanism: (i) *n* ≤ 0.43 suggests Fickian diffusion, where drug release is primarily governed by a concentration gradient; (ii) 0.43 < *n* < 0.85 indicates non-Fickian (anomalous) diffusion, where a combination of polymer swelling, chain relaxation, and diffusion influences drug release; and (iii) *n* ≥ 0.85 suggests case II transport, dominated by polymer relaxation and erosion. In this study, n values ranged from 0.61 to 0.65, suggesting that curcumin release followed a non-Fickian diffusion mechanism involving both polymer swelling and drug diffusion. This effect was more pronounced at pH 7.4, where polymer hydration and swelling were enhanced, facilitating faster release. The higher *K_KP_* values at pH 7.4 further support this, indicating greater polymer relaxation and diffusion-driven release. Conversely, at pH 5, the lower *K_KP_* values suggest a more restricted release, likely due to reduced polymer swelling in acidic conditions. The Weibull model is an empirical function that describes drug release kinetics, where parameter *a* represents the drug release rate and parameter *b* characterizes the release profile. The fact that all *b* values were below 0.75 confirms that the release mechanism is diffusion-controlled rather than being governed by burst release or erosion-driven processes. Differences in the a values across formulations indicate variations in release rates. Taken together, these findings indicate that the release mechanism is primarily governed by a balance of polymer swelling, diffusion, and relaxation, making the Korsmeyer–Peppas and Weibull models particularly suitable for describing the drug release behavior of these PHA-based nanoparticles.

### 2.3. Cytotoxicity and Antiproliferative Effects of PHA-Based Nanoparticles Loaded with Curcumin

The evaluation of cytotoxicity and antiproliferative activity is essential in determining the potential biomedical applications of nanoparticles. In this study, we assessed the cytotoxicity of PHA-based nanoparticles loaded with curcumin using the MTS assay on normal human astrocytes (NHAs) and fibroblasts. The antiproliferative effects were investigated on glioblastoma (U87 MG) and colon cancer (Caco-2) cell lines at different concentrations and time intervals.

Our results indicate that PHA-based nanoparticles exhibited no significant cytotoxicity on normal human astrocytes and fibroblasts, as cell viability remained above 90% even at the highest tested concentration of 40 μg/mL. These findings demonstrate the biocompatibility of the developed nanoparticles, in agreement with previous studies that reported the non-toxic nature of polyhydroxyalkanoates as nanoparticle matrices [8,9].

Meanwhile, the curcumin-loaded nanoparticles exerted an antiproliferative effect on U87 MG and Caco-2 cancer cells. After 24 h of treatment, a dose-dependent reduction in viability was observed for both U87 MG and Caco-2 cells. The inhibitory effects became more pronounced at 48 h at concentrations of 40 μg/mL. These results suggest that PHA-based nanoparticles effectively enhance the bioavailability and therapeutic efficacy of curcumin, supporting its potential use in cancer treatment.

**Figure 2:**
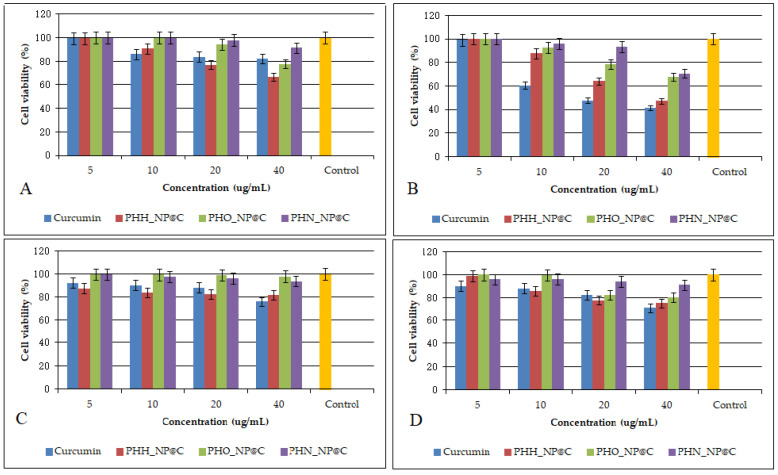
Antiproliferative effects of curcumin and PHA-based nanoparticles loaded with curcumin on U87 cells (**A**,**B**) and Caco-2 cells (**C**,**D**) after 24 and 48 h. Control is referring to untreated cells. Data are presented as the mean ± standard deviation. The results are considered statistically significant at *p* < 0.05.

Figure 2 presents the antiproliferative effects of curcumin and PHA-based nanoparticles on U87 (A, B) and Caco-2 (C, D) cells after 24 and 48 h. A clear dose-dependent decrease in cell viability is observed for both cell lines, particularly after 48 h, where higher concentrations (20 and 40 μg/mL) result in a reduction in viability. Curcumin-loaded nanoparticles exhibit cytotoxic effects emphasizing the improved delivery and sustained release properties of the nanosystem. This aligns with previous studies indicating that nanoparticles enhance the antiproliferative efficacy of curcumin by improving its bioavailability and intracellular accumulation [15]. The control samples maintain near 100% viability, confirming that the observed effects are due to the treatment. These findings further support the potential application of PHA-based nanoparticles for targeted cancer therapy. The IC_50_ values on U87 MG cells were 35.44 μg/mL (PHH_NP@C), 57.76 μg/mL (PHO_NP@C), 63.75 μg/mL (PHN_NP@C), and 24.87 μg/mL (free curcumin). The IC_50_ values on Caco-2 cells were 50.43 μg/mL (PHH_NP@C), 77.35 μg/mL (PHO_NP@C), 112.63 μg/mL (PHN_NP@C), and 81.67 μg/mL (free curcumin). Comparatively, previous research has demonstrated similar anticancer efficacy of polymeric nanoparticles loaded with curcumin. Hemmati et al. (2021) [30] reported IC₅₀ values of 5–30 μg/mL for U87 MG cells for chitosan-based curcumin nanosystems exhibiting higher cytotoxicity than our formulations. Senthilkumar et al. (2017) reported that polyhydroxybutyrate (PHB)-based nanoparticles loaded with curcumin exhibited sustained drug release and significant cytotoxicity against breast cancer cells through cell cycle arrest and apoptosis induction [23]. Likewise, Yallapu et al. (2010) showed that cyclodextrin–curcumin self-assembled nanoparticles enhanced curcumin delivery and exhibited strong antiproliferative effects on prostate cancer cells [25]. The results obtained in our study are consistent with these findings, further reinforcing the effectiveness of PHA-based nanoparticles in targeted cancer therapy.

Moreover, the observed antiproliferative effects align with studies on other biopolymeric carriers. Nanocarrier systems such as chitosan and alginate nanoparticles have demonstrated similar effects against Caco-2 cells, improving the solubility and cellular uptake of curcumin [18,19]. Pullulan acetate nanoparticles loaded with epirubicin, for example, demonstrated significant cytotoxicity against glioblastoma cells, with a similar time-dependent decrease in cell viability [27]. Our findings confirm that PHA-based nanoparticles provide a comparable and efficient drug delivery system for curcumin, with the advantages of biodegradability and biocompatibility.

A critical finding of this study is the selective cytotoxicity of PHA-based nanoparticles, as they exhibited negligible effects on normal human astrocytes and fibroblasts. This selectivity is crucial for minimizing adverse effects on healthy tissues and is consistent with other studies where polymeric nanoparticles preferentially accumulated in cancer cells, enhancing therapeutic efficacy while reducing systemic toxicity [25].

The cytotoxic effects observed in cancer cell lines treated with curcumin-loaded PHA nanoparticles can be attributed to curcumin’s well-documented mechanisms of action at the molecular level. Curcumin has been shown to induce apoptosis and cell cycle arrest through multiple pathways, including the phosphorylation of RAF-1, upregulation of p27 (Kip1), and downregulation of cyclins and cyclin-dependent kinases (CDKs), leading to G1/S or G2/M phase arrest [31,32,33]. Additionally, curcumin modulates key signaling pathways involved in cancer progression, such as the inhibition of NF-κB, AKT, and STAT3, as well as the activation of caspase-dependent apoptosis [31,33]. These mechanisms contribute to the enhanced cytotoxicity observed in glioblastoma (U87 MG) and colon cancer (Caco-2) cells in our study.

Furthermore, curcumin’s ability to induce oxidative stress and disrupt mitochondrial membrane potential has been reported as a contributing factor to its cytotoxic effects [32,33,34,35]. Since our study demonstrated a dose- and time-dependent reduction in cancer cell viability, it is likely that a combination of apoptosis induction, cell cycle arrest, and oxidative stress contributed to the observed antiproliferative effects. As a future perspective, additional mechanistic studies, such as Western blot analysis of apoptosis-related proteins (Bax, Bcl-2, caspases), cell cycle analysis by flow cytometry, and ROS detection assays, will be conducted to further elucidate the specific pathways involved in the cytotoxic effects of curcumin-loaded nanoparticles.

### 2.4. Evaluation of Dermal Toxicity and Irritability of PHA-Based Nanoparticles Loaded with Curcumin

The evaluation of dermal toxicity and irritability was conducted using the in vitro EpiDerm**™** reconstructed human skin model, a well-established alternative to animal testing, compliant with OECD TG 439 (Skin Irritation) and OECD TG 431 (Skin Corrosion) guidelines [36,37]. This model enables the assessment of the potential irritant and corrosive effects of pharmaceutical, cosmetic, and biomedical formulations while aligning with the 3R principle (Replacement, Reduction, and Refinement of animal testing). Given the growing demand for biocompatible and biodegradable nanocarriers, it was crucial to determine the skin compatibility of polyhydroxyalkanoate (PHA)-based nanoparticles encapsulating curcumin.

Following exposure of the EpiDerm**™** tissue inserts to the test nanoparticle formulations (PHH_NP@C, PHO_NP@C, PHN_NP@C) at a concentration of 25 μg/mL, MTT viability assays were performed to evaluate the metabolic activity of viable cells post-treatment. The data recorded following the test are presented in Table 6.

The results demonstrated that all tested formulations maintained cell viability above 85%, indicating the absence of significant cytotoxic effects. According to the classification criteria of the EU and GHS systems [38], a test substance is classified as irritant if the mean viability falls below 50% and as corrosive if viability drops below 15%. Given that all tested formulations exhibited viability well above these thresholds, they were classified as non-irritant and non-corrosive.

These results are in line with previous studies on biopolymeric nanoparticles, where PHAs were shown to possess excellent biocompatibility and low toxicity, making them suitable for drug delivery and dermal applications [39]. For example, Shrivastav et al. (2013) demonstrated that PHA nanoparticles exhibited no irritation effects in dermal fibroblast cultures, supporting their potential for topical formulations [39]. Similarly, Gregory et al. (2022) highlighted the non-toxic and biodegradable nature of PHAs, suggesting their safe use in wound-healing applications [9].

A statistical student *t* test further confirmed the safety profile of the nanoparticles. The mean optical density (OD) values of PHH_NP@C, PHO_NP@C, and PHN_NP@C were 1.189 ± 0.1301, 1.063 ± 0.1535, and 1.240 ± 0.188, respectively, with no statistically significant difference compared to the negative control (PBS, OD = 1.466 ± 0.1465, *p* > 0.05). In contrast, the positive control (1% SDS solution) resulted in a severe cytotoxic response with an OD of 0.057 ± 0.0063, confirming assay validity. The relative viability of PHH_NP@C, PHO_NP@C, and PHN_NP@C was 88.59%, 91.13%, and 92.41%, respectively, reinforcing their excellent skin compatibility.

Moreover, our findings align with studies on other biopolymeric carriers. For instance, chitosan- and alginate-based nanocarriers for curcumin have also been reported to exhibit minimal skin irritation, further supporting the biocompatibility of natural polymer-based nanoparticles [18,19]. Additionally, pullulan-based nanocarriers have been shown to possess excellent skin compatibility and enhanced drug permeation, making them promising for transdermal drug delivery [28]. These findings suggest that PHA-based nanoparticles represent a safe and effective nanocarrier system for dermal applications.

The results of this study indicate that PHA-based nanoparticles loaded with curcumin exhibit no potential for dermal irritation or corrosivity, confirming their safety for topical and transdermal applications in dermocosmetic, pharmaceutical, and medical device formulations. These findings are consistent with previous studies on biodegradable nanocarriers, reinforcing the potential of PHAs as non-toxic and biocompatible drug delivery systems. While the EpiDerm™ model provided valuable insights into the safety profile of PHA-based nanoparticles, additional studies on skin permeability and penetration depth are required to fully validate their potential for transdermal applications. Evaluating nanoparticle diffusion through the stratum corneum and deeper skin layers is crucial to determine their suitability for controlled drug delivery. Techniques such as Franz diffusion cell assays could provide quantitative data on nanoparticle permeation and localization within the skin. As a future perspective, we plan to conduct these permeability studies to strengthen the claim for dermal applicability and optimize nanoparticle formulations for transdermal drug delivery.

After conducting comprehensive in vitro tests (MTS assay for cytotoxicity and antiproliferative effects and EpiDerm™ assay for evaluation of dermal irritability and corrosivity), in vivo studies remain essential for fully assessing the therapeutic potential, safety, and pharmacokinetics of the developed formulation. While in vitro assays provide valuable insights into cytotoxicity, biocompatibility, and drug release, they do not fully replicate the complex physiological environment of living organisms. The dynamic interactions between nanoparticles and biological systems, including systemic circulation, metabolism, immune response, and tissue-specific accumulation, can only be evaluated through in vivo experiments.

Moreover, in vivo studies allow for the assessment of pharmacokinetic parameters such as drug absorption, distribution, metabolism, and excretion (ADME), which are crucial for determining the efficacy and safety of the formulation in a real biological system. Additionally, potential long-term effects, immunogenicity, and toxicity cannot be fully predicted by in vitro models alone. Therefore, in vivo validation is an indispensable step to bridge the gap between laboratory findings and clinical applications, ensuring the translation of research into effective therapeutic strategies. Therefore, our future research will focus on in vivo assessments to further validate their clinical applications in dermatology and regenerative medicine.

## 3. Materials and Methods

### 3.1. Materials

Curcumin and poly(ethylene-glycol)–block-poly(propylene-glycol)–block- poly(ethylene-glycol) (Pluronic F127) were purchased from Sigma-Aldrich (Merck Group, Darmstadt, Germany). Acetone and methanol were purchased from S.C. AdraChim S.R.L (Bucharest, Romania); human fibroblasts (ATCC-PCS-201-012), human glioblastoma U87 MG (ATCC-HTB-14), and colon cancer line Caco2 (ATCC-HTB-37) were acquired from ATCC; and NHAs were purchased from Lonza. Fetal equine serum (FES), Eagle’s minimum essential medium (EMEM), a mixture of penicillin, neomycin, and streptomycin dissolved in 0.9% NaCl (PSN), 0.25% trypsin–ethylene-diamine-tetraacetic-acid (EDTA) solution, Dulbecco’s Modified Eagle Medium/Nutrient Mixture F-12 (DMEM F12 Glutamax), N-2 Supplement, Fibroblast Growth Medium, Supplement Mix, sodium dodecyl sulfate 1% (SDS), and phosphate-buffered saline (PBS) were acquired from Sigma-Aldrich Co. (Merck Group, Darmstadt, Germany). An In Vitro EpiDerm™ Skin Irritation Test (EPI-200-SIT) was purchased from MatTek Co. (Bratislava, Slovak Republic). Polyhydroxyalkanoates (PHAs) with different compositions produced by bacterial fermentation with *Pseudomonas putida* ICCF 391 strain from the INCDCF-ICCF Collection of Microorganisms of Industrial Importance (CMII), affiliated with the World Federation of Culture Collections (WFCC), was chosen as the polymer matrix of the composites as presented previously [40,41,42]. The molecular weights of PHH, PHO, and PHN are 110.5 kDa, 141.8 kDa, and 165 kDa, respectively. Polyhydroxyheptanoat (PHH) had the highest content of 3-hydroxyheptanoic acid (87.46%), and polyhydroxyoctanoat (PHO) showed a predominant presence of 3-hydroxyoctanoic acid (85.93%), while polyhydroxynonanoat (PHN) was rich in 3-hydroxynonanoic acid (73.28%).

### 3.2. Preparation of Nanoparticles Based on PHAs Loaded with Curcumin

Curcumin-loaded polymeric nanoparticles were produced using the nanoprecipitation method. A polymer-to-drug solution at a 10:1 ratio was prepared by dissolving both components in 5 mL acetone serving as the organic phase (polymer concentration—10 mg/mL; drug concentration—1 mg/mL). This solution was introduced dropwise at a drop rate of 0.5 mL/min into an aqueous phase containing 1% Pluronic-F127 using a solvent-to-water phase ratio of 1:5 *v*/*v*, under continuous stirring at 700 rpm at room temperature. Complete evaporation of acetone under these conditions (700 rpm stirring rate, 72 h stirring time, room temperature) led to the formation of a nanosuspension. To isolate the nanoparticles, the suspension was subjected to centrifugation at 12,000 rpm for 20 min at 5 °C. The resulting nanoparticle precipitate was separated from the supernatant, which contained unentrapped curcumin, and subsequently redispersed in distilled water for further characterization.

### 3.3. Characterization of PHA-Based Nanoparticles Loaded with Curcumin

Dynamic light scattering (DLS) was used to determine particle size and polydispersity index using a Beckman Coulter N4 PCS Submicron instrument (Paris, France). Samples, diluted in double-distilled water (1:50), were analyzed at a fixed 90-degree angle at room temperature, with each measurement consisting of ten runs. Curcumin entrapment efficiency was indirectly evaluated via a UV/VIS spectrophotometer (Jasco V-630, Portland, OR, USA). The supernatant, diluted with methanol (0.25:10), was examined for curcumin absorbance at its maximum wavelength in methanol (421 nm) using a standard calibration curve (y = 0.0072 + 0.1137x; R^2^ = 0.99241). Stability testing involved evaluating entrapment efficiency after storing the nanoparticles in phosphate-buffered saline (PBS, pH 7.4) without stabilizers in amber glass vials at 4 °C for three months. Entrapment efficiency was assessed at 1, 2, and 3 months using a UV/VIS spectrophotometer. Additionally, nanoparticle aggregation was monitored via DLS measurements at each time point (1, 2, and 3 months) to evaluate changes in particle size.

### 3.4. In Vitro Curcumin Release from PHA-Based Nanoparticles

The in vitro release of curcumin from PHA-based nanoparticles was assessed using the dialysis bag method under optimized sink conditions. Dialysis membranes (Sigma-Aldrich, Merck Group, Darmstadt, Germany) with a molecular weight cut-off of 14,000 Da were used to enclose 0.5 mL of the nanoparticle suspension. These membranes were submerged in 100 mL of 0.1 M phosphate-buffered saline (PBS) at pH 5 and pH 7.4, maintained at 37 °C with continuous stirring at 150 rpm. To sustain sink conditions, the medium was periodically refreshed. Curcumin release was monitored at predetermined time points—5, 10, 15, 20, 25, 30, and 45 min, followed by 1, 2, 4, 5, 6, 24, 48, and 72 h—using a JASCO V-630 UV/VIS spectrophotometer (Jasco International Co., Ltd., Tokyo, Japan). Calibration curves for curcumin in PBS (0.1 M, pH 5 and pH 7.4) and ethanol were used to quantify the released compound. PBS at pH 5 was selected to simulate the slightly acidic tumor microenvironment, while PBS at pH 7.4 represented normal physiological pH.

### 3.5. In Vitro Cytotoxicity and Antiproliferative Effect of Nanoparticles Based on PHAs Loaded with Curcumin

The cytotoxicity and antiproliferative effects of curcumin-loaded PHA-based nanoparticles were evaluated using the MTS colorimetric assay. This assay is based on the use of a tetrazolium compound, [3-(4,5-dimethylthiazol-2-yl)-5-(3-carboxymethoxyphenyl)-2-(4-sulfophenyl)-2H-tetrazol] (MTS), and an electron coupling agent, phenazine methosulfate (PMS). Cells reduce the MTS reagent to a formazan product that is soluble in the culture medium. The absorbance of the formazan can be measured at 490 nm directly from 96-well culture plates. Human glioblastoma (U87 MG, ATCC-HTB-14), colon cancer (Caco2, ATCC-HTB-37), human astrocytes (NHA, Lonza), and fibroblasts (ATCC-PCS-201-012) were cultured under standardized conditions at 37 °C in a humidified 5% CO_2_ atmosphere. The growth medium varied depending on the cell type: DMEM F12 Glutamax supplemented with N-2 for astrocytes, fibroblast growth medium with a supplement mix for fibroblasts, and EMEM with 10% or 20% FBS for colon cancer cells. Once cultures reached 75% confluence, cells were detached using trypsin–EDTA, neutralized with fetal bovine serum, and centrifuged at 1200 rpm for 10 min. The resulting cell suspension was adjusted to a density of 10⁶ cells/mL and seeded into 96-well plates at 8000 cells per well. For the cytotoxicity assay, after 24 h of incubation, the medium was replaced with fresh medium containing PHA-based nanoparticles at concentrations of 5, 10, 20, and 40 µg/mL, and cells were exposed for 24 h at 37 °C with 5% CO_2_. Cell viability was assessed using a CellTiter 96^®^ AQueous One Solution Cell Proliferation Assay kit (Promega, San Luis Obispo, CA, USA). After 24 h of exposure to the aforementioned substance concentrations, the culture medium was replaced with 100 µL/well of MTS reagent, diluted 1:10 with fresh medium. The cells were incubated for 3 h with MTS reagent in the dark in a 5% CO_2_ incubator, after which the optical densities were measured at 495 nm using a microplate reader (Chameleon V Plate Reader, LKB Instruments, Victoria, Australia). Untreated cells served as controls, representing 100% viability. To evaluate the antiproliferative effect, U87 MG and Caco2 tumor cells were treated with the same nanoparticle concentrations, but exposure times were extended to 24 and 48 h at 37 °C with 5% CO_2_. Viability was measured at these time points after one population-doubling interval. Untreated cells were used as the baseline control. The tests were performed in triplicate.

### 3.6. Evaluation of Dermal Irritability and Corrosivity of Nanoparticles Based on PHAs Loaded with Curcumin

The irritability and corrosivity of PHH, PHO, and PHN nanoparticles were evaluated following the In Vitro EpiDerm™ Skin Irritation Test (EPI-200-SIT) protocol, provided by MatTek (Bratislava, Slovak Republic), a distributor of in vitro kits from the American company MatTek. A reconstituted human skin model, consisting of 24 viable tissue inserts embedded in agarose and supplemented with DMEM (10% fetal serum), was used. The positive control was 1% sodium dodecyl sulfate (SDS) in phosphate-buffered saline (PBS).

#### 3.6.1. Tissue Pre-Cultivation

Eight 6-well plates were pre-incubated with 0.9 mL of culture medium per well at 37 °C, 5% CO_2_, for 30 min. Tissue inserts were rinsed with PBS, placed in wells, and incubated overnight.

#### 3.6.2. Sample Preparation and Exposure

SDS (positive control) was prepared in PBS, while test samples (PHH_NP@C, PHO_NP@C, PHN_NP@C) were suspended in DMEM. Each sample (25 μg) was applied per well. After incubation at 37 °C for 35 ± 1 min and an additional 25 ± 1 min in a laminar flow hood, tissues were rinsed 20 times with PBS and incubated in fresh medium for 24 ± 2 h, followed by 18 ± 2 h.

In this study, we selected a concentration of 25 µg/mL for testing in the EpiDerm™ model based on the existing literature [42,43] and OECD guidelines for in vitro skin irritation and corrosion assays [36]. The concentration used in this study ensures that potential cytotoxic and irritant effects can be detected without exceeding the viability thresholds required for reliable assay performance. Additionally, it reflects typical exposure levels used in preliminary safety assessments for nanomaterials in dermal applications. While higher doses could provide further insights into potential dose-dependent effects, excessively high nanoparticle concentrations may lead to non-specific aggregation or altered diffusion, affecting the accuracy of toxicity evaluations. The OECD Test Guideline No. 439 outlines the use of reconstructed human epidermis models, like EpiDerm™, for in vitro skin irritation testing. While it does not specify exact concentrations for all test substances, it emphasizes using doses that maintain tissue viability and barrier function, ensuring the reliable assessment of irritancy [31]. Casas et al. used the EpiDerm™ model to assess the skin irritation potential of medical device polymers, and their findings highlight the EpiDerm™ model’s capability to detect strong skin irritants at low concentrations in medical device extract mixtures [42]. Also, Wills et al. employed nanoparticle concentrations ranging from 150 µg to 450 µg per tissue for topical exposures. These doses were selected to balance effective exposure with tissue viability, avoiding non-specific aggregation that can occur at higher concentrations. Future studies will explore a broader concentration range, including therapeutically relevant doses, to better simulate real-world dermal exposure conditions.

#### 3.6.3. MTT Assay and Viability Assessment

Following incubation, tissues were transferred to a 24-well plate, and culture medium was aspirated for cytokine analysis (IL-1β, TNF-α). Tissues were incubated with MTT reagent at 37 °C, 5% CO_2_, for 3 h, allowing viable cells to convert MTT into formazan. The formazan was solubilized with acidified isopropanol and incubated for 2 h on a shaker. Optical density (OD) at 570 nm was measured using a microplate reader. Relative viability was calculated from OD values of test samples, negative controls, and positive controls, classifying test substances based on a Predictive Model. According to the Predictive Model based on EU and GHS classification (Category R38/Category 2 or no labelling) [38], a substance is considered an irritant if the mean tissue viability of three individual tissues exposed to the test substance is below 50% relative to the negative controls. If the mean tissue viability is below 15% relative to the negative controls, the product is classified as corrosive. The tests were conducted in triplicate.

## 4. Conclusions

The polymeric nanoparticles based on polyhydroxyalkanoates (PHAs) loaded with curcumin were successfully synthesized via the nanoprecipitation method using biopolymeric matrices with different compositions obtained from *Pseudomonas putida* fermentation. The developed nanoparticles exhibited high entrapment efficiency (above 80%), nanometric size, narrow size distribution, and excellent stability, maintaining their structural integrity for up to three months at 4 °C. The in vitro release studies demonstrated a controlled release profile, influenced by the PHA composition, with a slower release in acidic conditions (pH 5), relevant for tumor microenvironments. The cytotoxicity assays confirmed that the nanoparticles were biocompatible, as they did not induce toxicity in normal human astrocytes (NHAs) and fibroblasts. Furthermore, the antiproliferative studies on glioblastoma (U87 MG) and colon cancer (Caco-2) cell lines revealed that the curcumin-loaded nanoparticles effectively reduced cancer cell viability in a dose- and time-dependent manner, enhancing the therapeutic potential of curcumin. The dermal toxicity and irritation assessments, conducted using the EpiDerm™ model, demonstrated that the nanoparticles were non-irritant and non-corrosive, confirming their suitability for dermal and transdermal applications. These findings align with previous studies on biopolymer-based nanocarriers, reinforcing the safety and efficacy of PHAs as drug delivery systems. In conclusion, PHA-based nanoparticles loaded with curcumin represent a promising and biocompatible drug delivery platform with potential applications in cancer therapy, regenerative medicine, and dermocosmetic formulations. While our study demonstrates the biocompatibility of PHA-based nanoparticles through MTS cytotoxicity assays, further validation is necessary to fully confirm their safety profile. In particular, additional studies using flow cytometry-based apoptosis assays (Annexin V/PI staining) and ROS detection assays would provide deeper insights into the mechanisms of cell viability and oxidative stress induction. These complementary assays will help assess whether the nanoparticles induce apoptotic or necrotic pathways and confirm the absence of oxidative stress-related toxicity. As a future perspective, we plan to conduct these analyses to further substantiate the biocompatibility of the developed nanoparticles.

Further in vivo studies are necessary to validate these findings and to gain deeper insights into the pharmacokinetics, bioavailability, and therapeutic efficacy of the nanoparticles in a living system, such as laboratory animals (e.g., mice, rats, rabbits). As a future perspective, we plan to extend our research to in vivo models to evaluate the nanoparticles’ systemic distribution, toxicity, and tumor-targeting capabilities. These investigations will be essential for the translational potential of our proposed drug delivery system. Additionally, further optimization of formulation parameters, such as particle size, surface modifications, and drug loading efficiency, is necessary to enhance their stability, targeting ability, and bioavailability.

## Figures and Tables

**Table 1:** Features of nanoparticles based on PHAs loaded with curcumin.

Sample	EE (%)	Size (nm) *	PDI
PHH_NP@C	80.41 ± 1.25	307.5 ± 3.44	0.219 ± 1.24
PHO_NP@C	82.47 ± 0.11	309.9 ± 1.55	0.247 ± 1.79
PHN_NP@C	84.35 ± 0.23	315 ± 2.76	0.289 ± 1.34

* Size (nm) represents the mean hydrodynamic diameter of the nanoparticles, expressed in nanometers (nm).

**Table 2:** Stability of nanoparticles based on PHAs loaded with curcumin at 4 °C—curcumin content evaluation.

Sample	Curcumin Content (mg per Total Formulation)			
	Initial	1 Month	2 Months	3 Months
PHH_NP@C	4.12 ± 0.01	4.10 ± 0.02	4.08 ± 0.04	4.04 ± 0.02
PHO_NP@C	4.21 ± 0.02	4.20 ± 0.03	4.18 ± 0.03	4.13 ± 0.03
PHN_NP@C	4.25 ± 0.01	4.23 ± 0.02	4.21 ± 0.01	4.17 ± 0.04

**Table 3:** Stability of nanoparticles based on PHAs loaded with curcumin at 4 °C—DLS evaluation.

Sample	Size (nm)			
	Initial	1 Month	2 Months	3 Months
PHH_NP@C	307.5 ± 3.44	308.4 ± 5.84	309.1 ± 3.54	310.5 ± 1.44
PHO_NP@C	309.9 ± 1.55	310.5 ± 2.55	311.2 ± 2.65	311.9 ± 2.45
PHN_NP@C	315 ± 2.76	315.8 ± 3.75	316.3 ± 1.66	317 ± 2.44

**Table 4:** Kinetic parameters (R^2^, AIC, and RMSE) for zero-order, first-order, Korsmeyer–Peppas, Higuchi, Hixson–Crowell, Weibull, and Baker–Lonsdale models.

	Release Medium: PBS 0.1 M pH 7.4			Release Medium: PBS 0.1 M pH 5		
Model	R^2^	AIC	RMSE	R^2^	AIC	RMSE
PHH_NP@C						
Zero order	0.7768	106.1746	10.2735	0.9077	78.6231	3.8404
First order	0.1436	156.9316	62.9483	0.2030	134.8438	28.6016
Hixson–Crowell	0.4299	118.3895	15.8919	0.6595	87.9145	5.3517
Higuchi	0.6695	111.6706	12.5015	0.5684	100.2178	8.3047
Korsmeyer–Peppas	0.9741	114.3055	13.7351	0.9887	84.1526	4.6789
Weibull	0.9744	91.5702	6.0981	0.9865	86.8556	5.1531
Baker–Lonsdale	0.9624	145.5648	41.9449	0.7769	123.1996	18.8705
PHO_NP@C						
Zero order	0.6454	111.2418	12.3115	0.8576	94.3508	6.7348
First order	0.1283	157.8511	65.0497	0.1355	144.6726	40.6294
Hixson–Crowell	0.3055	121.0583	17.4812	0.4978	103.7460	9.4200
Higuchi	0.3839	118.9761	16.2283	0.8221	97.4691	7.5282
Korsmeyer–Peppas	0.9387	126.2095	21.0121	0.9943	108.8406	11.2997
Weibull	0.9384	109.7094	11.6558	0.9959	92.2845	6.2557
Baker–Lonsdale	0.9443	148.4218	46.4508	0.8917	138.6672	32.7864
PHN_NP@C						
Zero order	0.5499	113.3368	13.2680	0.9273	87.4120	5.2565
First order	0.1161	154.0049	56.7008	0.1436	147.6702	45.2204
Hixson–Crowell	0.2615	120.5077	17.1408	0.5627	102.6912	9.0717
Higuchi	0.2499	120.4888	17.1292	0.8504	97.5217	7.5423
Korsmeyer–Peppas	0.9466	131.6434	25.5124	0.9964	102.2520	8.9305
Weibull	0.9458	116.8895	15.0629	0.9980	80.4967	4.1062
Baker–Lonsdale	0.8197	148.3988	46.4126	0.9513	139.8785	34.2359

**Table 5:** Kinetic parameters of the Korsmeyer–Peppas and Weibull models.

		Korsmeyer–Peppas Parameters		Weibull Parameters	
Sample	Release Medium	*n*	*K_KP_*	*a*	*b*
PHH_NP@C	PBS 0.1 M pH 7.4	0.6503	31.2864	2.4498	0.6663
PHO_NP@C	PBS 0.1 M pH 7.4	0.6555	35.1844	2.083	0.6727
PHN_NP@C	PBS 0.1 M pH 7.4	0.6534	37.1902	1.9703	0.6700
PHH_NP@C	PBS 0.1 M pH 5	0.6103	10.5847	8.6340	0.6162
PHO_NP@C	PBS 0.1 M pH 5	0.6360	26.5643	3.1105	0.6475
PHN_NP@C	PBS 0.1 M pH 5	0.6370	26.9083	3.0422	0.6493

**Table 6:** Dermal irritation and corrosivity evaluation of PHA-based nanoparticles (EpiDerm™ model).

Product	Mean OD	STDEV	P ^(3)^	P ^(4)^	Relative Viability (%)
SDS, ^1^	0.057	0.0063	-	-	1.92
PBS, ^2^	1.466	0.1465	-	-	100
PHH_NP@C	1.189	0.1301	2.9 × 10^−9^	0.056	88.59
PHO_NP@C	1.063	0.1535	1.3 × 10^−8^	0.166	91.13
PHN_NP@C	1.240	0.188	6.4 × 10^−8^	0.285	92.41

^1^—positive control; ^2^—negative control; ^3^—Student *t* test, 2 tails, homoscedastic distribution, compared to negative control; ^4^—Student *t* test, 2 tails, homoscedastic distribution, compared to positive control; OD—optical density.

## Data Availability

Data to support statements in this article are available from the corresponding author [Pavaloiu Ramona-Daniela], upon reasonable request.
